# Antibacterial activity and mechanisms of D-3263 against *Staphylococcus aureus*

**DOI:** 10.1186/s12866-024-03377-3

**Published:** 2024-06-26

**Authors:** Xiaoju Liu, Yanpeng Xiong, Renhai Peng, Yufang Zhang, Shuyu Cai, Qiwen Deng, Zhijian Yu, Zewen Wen, Zhong Chen, Tieying Hou

**Affiliations:** 1 Department of Infectious Diseases, Shenzhen Key Laboratory for Endogenous Infection, Huazhong University of Science and Technology Union Shenzhen Hospital, No 89, Taoyuan Road, Nanshan District, Shenzhen, 518052 China; 2 Department of Infectious Diseases and Shenzhen key Laboratory of Endogenous infection, Shenzhen Nanshan People’s Hospital and the 6th Affiliated Hospital of Shenzhen University Medical School, No 89, Taoyuan Road, Nanshan District, Shenzhen, 518052 China; 3 Department of Biology, Washington University in St. Louis, 1 Brookings Drive, St Louis, MO 63130 USA

**Keywords:** Antibacterial activity, Biofilm, D-3263, Phospholipids, *Staphylococcus aureus*

## Abstract

Multi-drug-resistant *Staphylococcus aureus* infections necessitate novel antibiotic development. D-3263, a transient receptor potential melastatin member 8 (TRPM8) agonist, has potential antineoplastic properties. Here, we reported the antibacterial and antibiofilm activities of D-3263. Minimum inhibitory concentrations (MICs) against *S. aureus*, *Enterococcus faecalis* and *E. faecium* were ≤ 50 µM. D-3263 exhibited bactericidal effects against clinical methicillin-resistant *S. aureus* (MRSA) and *E. faecalis* strains at 4× MIC. Subinhibitory D-3263 concentrations effectively inhibited *S. aureus* and *E. faecalis* biofilms, with higher concentrations also clearing mature biofilms. Proteomic analysis revealed differential expression of 29 proteins under 1/2 × MIC D-3263, influencing amino acid biosynthesis and carbohydrate metabolism. Additionally, D-3263 enhanced membrane permeability of *S. aureus* and *E. faecalis*. Bacterial membrane phospholipids phosphatidylethanolamine (PE), phosphatidylglycerol (PG), and cardiolipin (CL) dose-dependently increased D-3263 MICs. Overall, our data suggested that D-3263 exhibited potent antibacterial and antibiofilm activities against *S. aureus* by targeting the cell membrane.

**Supplementary Information:**

The online version contains supplementary material available at 10.1186/s12866-024-03377-3.

## Introduction

*S. aureus* is a Gram-positive bacterium responsible for a wide spectrum of infections, both within the community and healthcare settings [1]. This versatile pathogen often resides on the human skin and in the nasopharynx, and once it gains access to the internal human body, it can trigger infections affecting various anatomical sites, encompassing skin, respiratory tract, bloodstream, endocardium, and cardiac chambers and valves [2, 3]. One of the major challenges in treating *S. aureus* infections is the emergence of antibiotic-resistant strains. Clinical isolates have acquired resistance to many beta-lactam antibiotics, including penicillin and methicillin, through the acquisition of the *mecA* gene, which encodes the altered penicillin-binding protein 2a (PBP 2a) [4]. These strains are commonly known as methicillin-resistant *S. aureus* (MRSA) due to their resistance to methicillin, the first beta-lactamase-stable penicillin derivative introduced into clinical practice. Furthermore, *S. aureus* has exhibited diminished susceptibility or resistance to several reserved antibiotics, including but not limited to vancomycin, linezolid, and daptomycin [5, 6]. The rise of multidrug-resistant *S. aureus*, particularly MRSA, has become a grave concern in healthcare settings. These resistant strains pose a significant challenge to effective treatment, often leading to prolonged hospital stays and increased healthcare costs [1]. Consequently, there is an urgent need to develop innovative and effective antibiotic treatments to combat MRSA and other drug-resistant *S. aureus* strains [7].

*S. aureus*’ capacity to adhere to and establish bacterial biofilms on the surfaces of implanted medical devices, such as cardiac pacemakers, artificial heart valves, and joint replacements, poses a formidable challenge in clinical practice [8, 9]. Biofilm formation by this bacterium significantly complicates treatment, as it bestows heightened resistance to antibiotics and host immune responses. This, in turn, often leads to the development of chronic infections characterized by recurrent symptoms [10–12]. Biofilm-associated infections, particularly those caused by staphylococci, represent a prevalent and persistent clinical concern. Among the staphylococci, *S. aureus* stands out as one of the most prevalent etiologic agents responsible for device-related infections. These infections substantially elevate treatment costs, extend hospitalization duration, and contribute to increased morbidity and mortality rates [8, 13]. Consequently, it is imperative for the scientific community to prioritize the development of strategies aimed at targeting *S. aureus* biofilm formation and effectively eliminating established biofilms. Such efforts are crucial in the ongoing quest for novel antibiotics and therapeutic interventions to address the complex challenges posed by biofilm-associated infections.

Similar to *S. aureus. E. faecalis* and *E. faecium*, members of the *Enterococcus* genus, are also widely prevalent Gram-positive pathogens globally in healthcare settings [14]. *E. faecalis* and *E. faecium* can cause a wide range of infections in humans, including urinary tract infections, wound infections, intra-abdominal infections, endocarditis, and bloodstream infections. Enterococci species exhibit intrinsic resistance to multiple antibiotics, such as cephalosporins, aminoglycosides, lincosamides, and sulfonamides. Additionally, due to the genetic plasticity of their genomes, enterococci can acquire new resistance determinants through horizontal gene transfer. Vancomycin-resistant enterococci (VRE) carrying van genes, such as vanA cluster, have emerged as one of the major multidrug-resistant nosocomial bacteria worldwide [15].

**Fig. 1:**
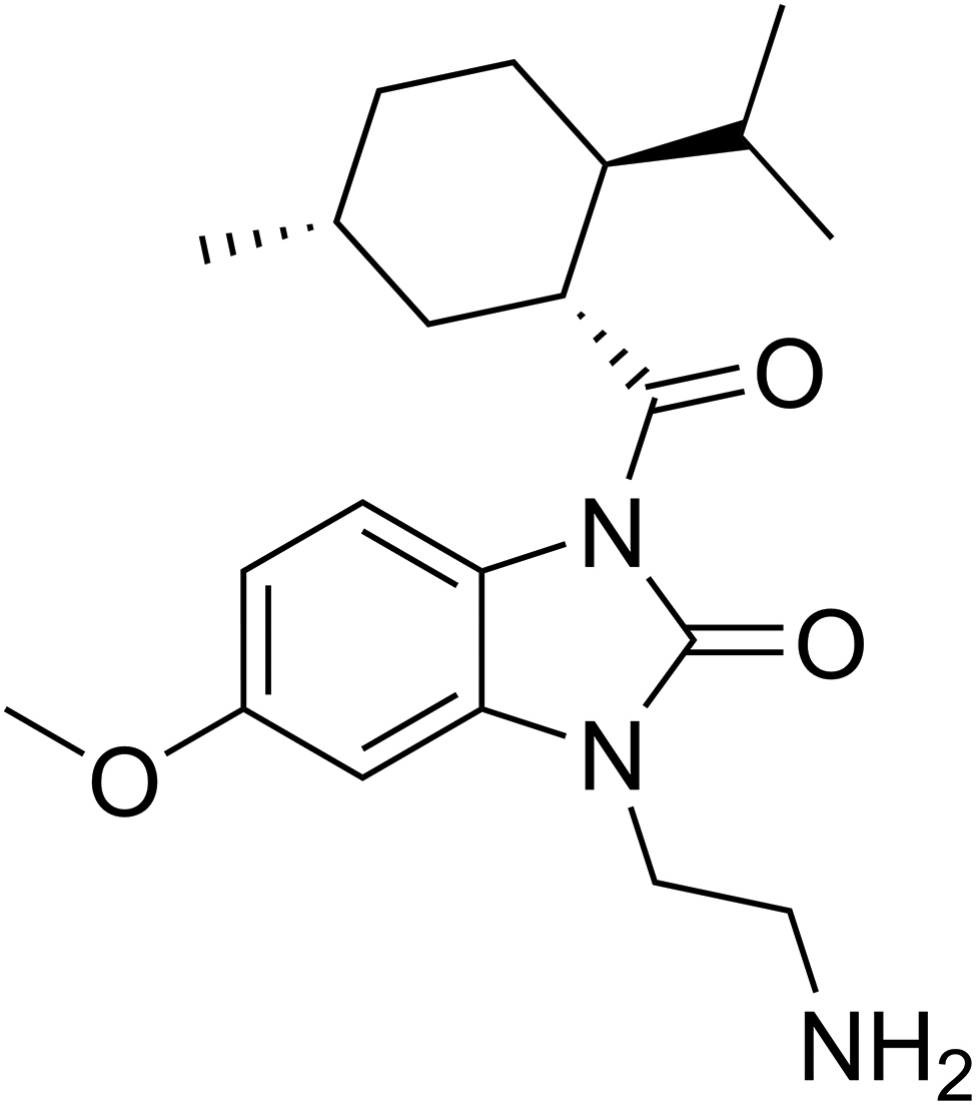
Molecular structure of D-3263. The chemical formula of D-3263 is C21H32ClN3O3

D-3263 hydrochloride (Fig. 1), an orally administered, enteric-coated compound that has successfully completed phase 1 clinical trials focused on dosage and pharmacokinetics [16], holds promise as an agonist targeting transient receptor potential melastatin member 8 (TRPM8 or Trp-m8). Initially cloned from the prostate gland, TRPM8 has since been detected in various neuronal and non-neuronal tissues and organs, and has emerged as a significant player in the context of various life-threatening tumors, including melanoma, breast cancer, pancreatic cancer, and others [17, 18]. In a notable study focusing on metastatic prostate cancer, D-3263 exhibited the ability to induce apoptotic responses in aggressive mouse prostate adenocarcinomas [19]. The proposed mechanism underlying this phenomenon involves D-3263 triggering a gradual elevation of intracellular Ca^2+^ levels in mouse prostate adenocarcinomas cells. As research into TRPM8 and its pharmacological modulation by compounds like D-3263 advances, there is growing excitement about the potential therapeutic applications, particularly in the context of cancer treatment. In this paper, we first reported on the antimicrobial performance of D-3263, assessing its effect on gram-positive bacteria *S. aureus*, *E. faecium* and *E. faecalis*. The inhibitory and eliminating effect of D-3263 against both planktonic cells and biofilms were evaluated in this study, and its mode of action was then explored using checkerboard assay with phospholipids and mass spectrum-based quantitative proteomics.

## Results

### In vitro antimicrobial activity of D-2363 against Gram-positive bacteria

Minimum inhibitory concentrations (MICs) of D-2363 against 30 clinical *S. aureus* isolates were tested using the broth dilution method in 96 well plates. The 30 clinical *S. aureus* isolates included 15 methicillin-sensitive *S. aureus* (MSSA) and 15 methicillin-resistant *S. aureus* (MRSA), corresponding test results are listed in Table 1. The results suggested D-3263 possessed a significant inhibitory effect against *S. aureus* isolates, generally at 25 µM for both MSSA (86.67%) and MRSA (93.33%). D-3263 was also discovered to have inhibitory effect on *E. faecalis* and *E. faecium*. MICs of D-3263 against clinical *E. faecalis* and *E. faecium* isolates were found to be ≤ 25 µM for all 20 clinical isolates used. In addition, we tested the inhibitory activity of D-3263 against several clinically common Gram-negative bacteria, including *Escherichia coli*, *Acinetobacter baumannii*, *Pseudomonas aeruginosa*, and *Klebsiella pneumoniae*. The results showed that the MIC values were all greater than 200 µM (Table S1), indicating that D-3263 only exhibits inhibitory activity against Gram-positive bacteria.

**Table 1:** MIC distribution of D-3263 against Gram-positive bacteria

Class	No. of isolates tested	MIC distributiong		
		D-3263 MIC = 12.5 µM	D-3263 MIC = 25 µM	D-3263 MIC = 50 µM
MSSA	15	1 (6.67%)	13 (86.67%)	1 (6.67%)
MRSA	15	1 (6.67%)	14 (93.33%)	-
*E. faecalis*	10	-	10 (100%)	-
*E. faecium*	10	1 (10%)	9 (90%)	-

MIC, Minimum inhibitory concentration; MSSA, methicillin-sensitive S. aureus; MRSA, methicillin-resistant *S. aureus*; No., number

**Fig. 2:**
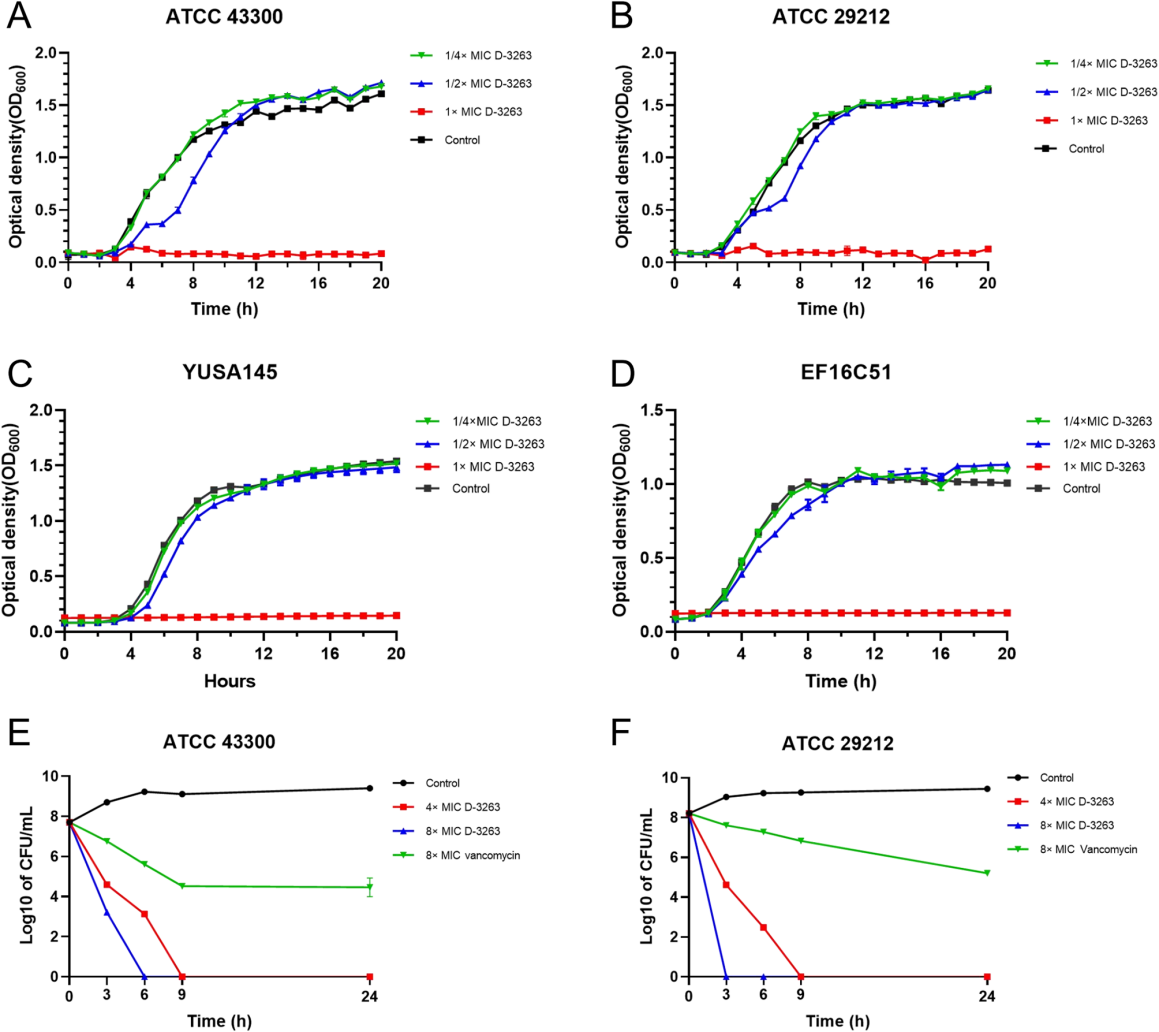
In vitro antimicrobial activity of D-2363 against Gram-positive bacteria. Two reference strains (MRSA ATCC 43,300 and *E. faecalis* ATCC 29,212) (**A, B**) and two clinical isolates (*S. aureus* YUSA145 and *E. faecalis* EF16C51) (**C, D**) were included in the analysis. The strains were individually cultured at different concentrations of D-3263, including 1× MIC, 1/2× MIC, and 1/4× MIC. The MICs for all strains used in this assay were 25 µM. Absorbance at a wavelength of 600 nm (OD_600_) was measured at 1 h intervals for a total duration of 20 h. The bactericidal effect of D-3263 against *S. aureus* and *E. faecalis*. MRSA ATCC 43,300 (**E**) and *E. faecalis* ATCC 29,212 (**F**), cultured to the exponential phase, were exposed to D-3263 at concentrations of 4×, and 8× MIC, as well as 8× MIC of vancomycin for comparison. Samples were collected and counted at 0, 3, 6, 9, and 24 h. The experiments were conducted in triplicate, and the results were presented as mean ± SEM.

The effect of D-3263 at subinhibitory concentrations on the growth of *S. aureus* planktonic cells was evaluated using the reference MRSA ATCC 43,300 and the clinical MRSA isolate YUSA145. As illustrated in Fig. 2A and C, D-3263 at a concentration of 1× MIC completely suppressed the growth of the tested bacterial planktonic cells. At 1/2× MIC, D-3263 exhibited a modest inhibitory effect, slowing down planktonic cell growth, but it was unable to halt the cells from reaching their maximum growth rate during the stationary phase after 20 h. A similar growth-slowing effect of D-3263 at 1/2× MIC was observed against *E. faecalis* using reference strain ATCC 29,212 and clinical isolate EF16C51, as shown in Fig. 2B and D.

The bactericidal activity of D-3263 was assessed using the microdilution method. The MBC values of D-3263 against *S. aureus* ATCC 29,213 and ATCC 43,300 strains were found to be 25 µM, which are equivalent to the MIC values (Table 2). The MBC values of D-3263 against *E. faecalis* ATCC 29,212 was 50 µM, which was 2-fold of the MIC. The MBC of all the measured strains was less than 4 times the corresponding MIC value, indicating that D-3263 was a bactericidal agent. The bactericidal efficacy of D-3263 against *S. aureus* and *E. faecalis* planktonic cells was further assessed and compared to linezolid using a time-killing assay, with MRSA ATCC 43,300 and *E. faecalis* ATCC 29,212 as test strains (Fig. 2E-F). After 6 h, our data revealed that while linezolid exhibited no bactericidal effect, D-3263 at both 4× and 8× MIC demonstrated a significant bactericidal effect against *S. aureus*, as evidenced by a reduction in the number of viable cells. Specifically, D-3263 at 8× MIC exhibited a notably stronger bactericidal effect against *E. faecalis*, reducing cell counts to the lower limit of detection within 6 h. After 24 h, D-3263 at 4× MIC had completely eradicated all *E. faecalis* cells, while the number of *S. aureus* cells treated with 8× MIC was further reduced, although not completely eliminated. These results underscored the potent bactericidal activity of D-3263 against both *S. aureus* and *E. faecalis*.

**Table 2:** MIC and MBC for D-3263 against *S. aureus* and *E. faecalis* (µM)

	Strains	MIC	MBC
	ATCC29213	25	25
	ATCC43300	25	25
*S. aureus*	SA113	25	25
	YUSA145	25	50
	USA300	25	25
	ATCC29212	25	50
*E. faecalis*	OG1RF	25	25
	FB-1	25	25

### Anti-biofilm activity of D-3263

**Fig. 3:**
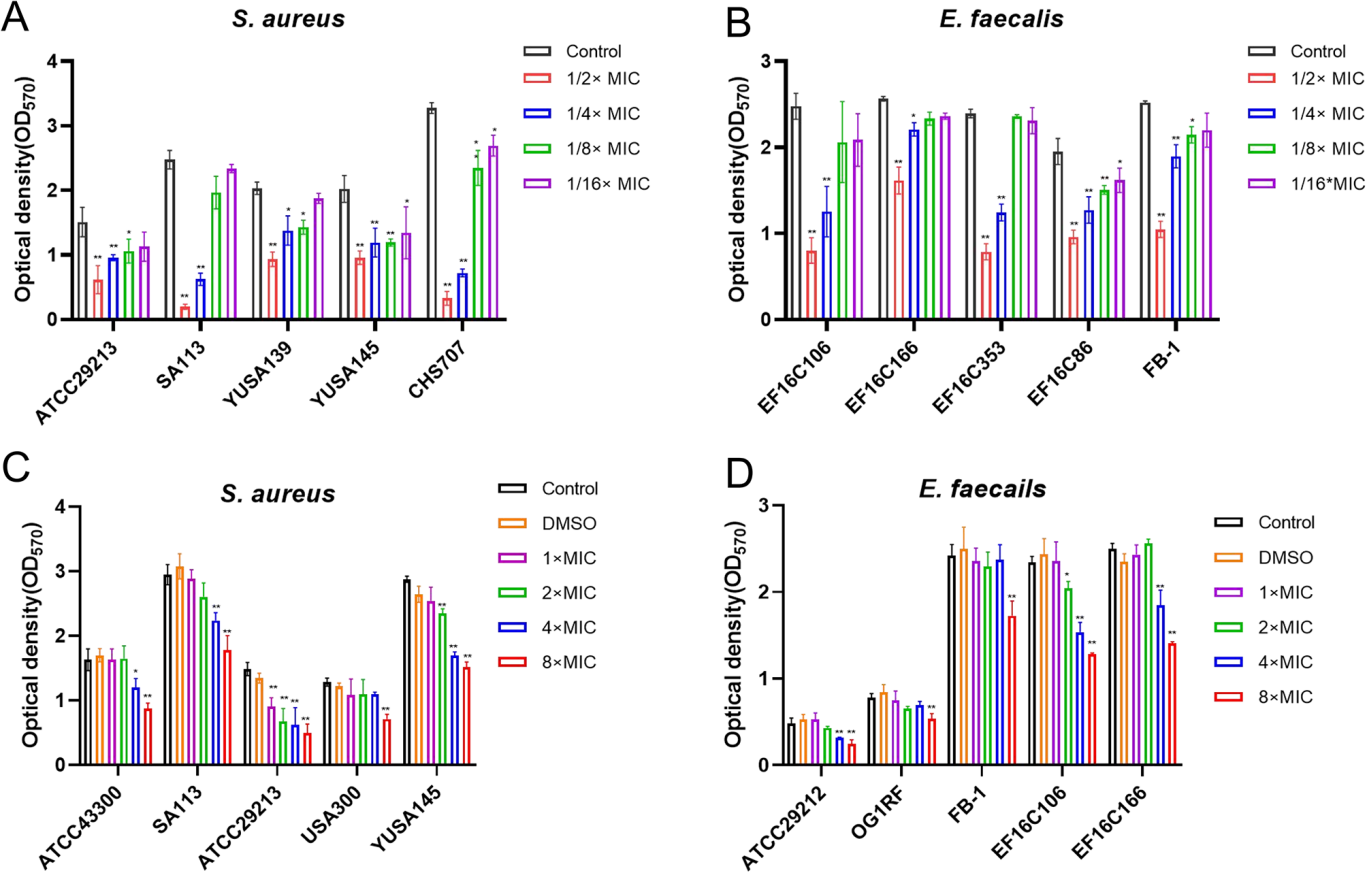
Anti-biofilm activity of D-3263. Five *S. aureus* isolates, which included three clinical MRSA isolates (YUSA139, YUSA145, and CHS707) (**A**), along with five *E. faecalis* strains (**B**), were subjected to a 24-hour treatment with D-3263 at concentrations of 1/2×, 1/4×, 1/8×, and 1/16× MIC. Following treatment, crystal violet staining was employed to visualize and assess the biofilm formed. The biofilm biomass was semi-quantitatively measured by determining the OD_570_. (**C, D**) The effect of D-3263 on mature *S. aureus* and *E. faecalis* biofilm. five E. *faecalis* and five *S. aureus* isolates with mature biofilms were exposed to D-3263 at concentrations of 8×, 4×, 2×, and 1× MIC. Control represents the biofilm before the addition of the compound, while the DMSO group represents the solvent-treated group. The data were presented as the average of three independent experiments (mean ± SEM). Statistical significance was determined using Student’s *t*-test, where * indicates *P* < 0.05 and ** indicates *P* < 0.01 compared to the control group

Anti-biofilm activity of D-3263 was assessed by examining its inhibitory effect on biofilm formation in five *S. aureus* isolates and five *E. faecalis* isolates. The biofilm biomass was quantified using crystal violet staining after treatment with subinhibitory concentrations of D-3263. As depicted in Fig. 3A-B, D-3263 markedly inhibited biofilm formation in all tested *S. aureus* and *E. faecalis* strains at concentrations ranging from 1/2× to 1/4× MIC. These findings underscored the potent anti-biofilm properties of D-3263.

Following the demonstration of D-3263’s capability to inhibit biofilm formation, we conducted further investigations to assess its effectiveness in eradicating mature biofilms formed by *S. aureus* and *E. faecalis*. Different concentrations of D-3263, ranging from 1× MIC to 8× MIC, were introduced to preformed bacterial biofilms. As depicted in Fig. 3C-D, remarkable biofilm reduction was observed at 8× MIC for all *S. aureus* and *E. faecalis* isolates, and at 4× MIC, significant reduction was evident in 7 out of 10 tested isolates. The above results indicated that D-3263 possessed anti-biofilm activity against Gram-positive bacteria.

### **Proteomic analysis of the global response of** *S. aureus* **to D-3263 stress**

**Fig. 4:**
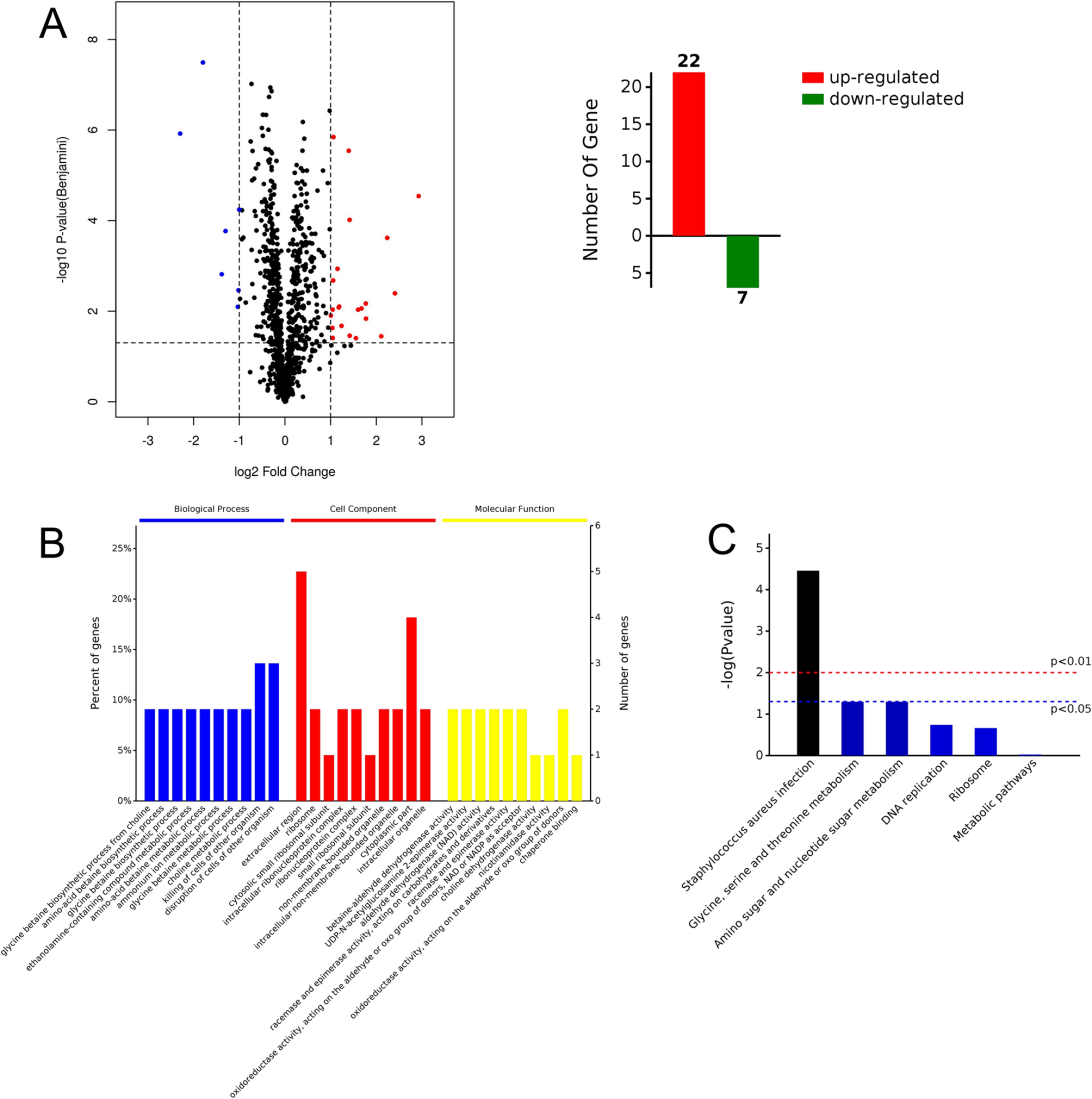
Proteomic analysis of the global response of *S. aureus* to D-3263 stress. (**A**) The volcano map displays the differentially expressed proteins, with red representing up-regulated proteins and blue representing down-regulated proteins. The horizontal axis represents the ratio of differentially expressed proteins between the D-3263 treated and control groups, while the vertical axis represents the *p*-value comparing the two groups. (**B**) The bar chart shows the number of up and down-regulated proteins. (**C**) Gene Ontology (GO) analysis was performed to categorize the differentially expressed proteins based on their involvement in biological process, cell component, and molecular function. (**D**) Kyoto Encyclopedia of Genes and Genomes (KEGG) pathway analysis was conducted to identify the pathways associated with the differentially expressed proteins

Quantitative label-free proteomic analysis was conducted to investigate the proteomic response of *S. aureus* when subjected to D-3263 stress. Following a 2-hour treatment with 1/2× MIC of D-3263 (12.5 µM), the protein expression profile of the clinical MRSA isolate YUSA145 was extracted for quantitative proteomic analysis. PCA and heatmap data revealed global differences in the protein profiles between the D-3263 treatment group and the control group (Fig. S1). Totally, 29 proteins exhibited significant expression changes (≥|2.0|-fold change, *p* ≤ 0.05), with 22 of them being up-regulated and 7 being down-regulated in comparison to the DSMO control group (Fig. 4A-B; Table 2).

Subsequently, a Gene Ontology (GO) enrichment analysis was conducted to elucidate the biological processes, cell component and molecular functions in which the differentially expressed proteins were implicated (Fig. 4C). This analysis revealed significant enrichment in processes related to the destruction and killing of cells from other organisms, as well as metabolic processes involved with ammonium ions, choline, amino-acid betaine, glycine betaine, and ethanolamine-containing compounds. Additionally, biosynthetic processes for amino-acid betaine, glycine betaine, and the synthesis of glycine betaine from choline were significantly enriched. Furthermore, a Kyoto Encyclopedia of Genes and Genomes (KEGG) pathway enrichment analysis was performed [20], indicating that the differentially expressed proteins in the D-3263 treatment group were significantly enriched in virulence pathways related to *S. aureus* infection (Fig. 4D), including Bi-component leukocidin LukGH subunit H, Immunoglobulin-binding protein Sbi, Bi-component leukocidin LukGH subunit G, MAP domain-containing protein and Gamma-hemolysin component B.

### D-3263 targets phospholipids to exert antimicrobial activity

**Fig. 5:**
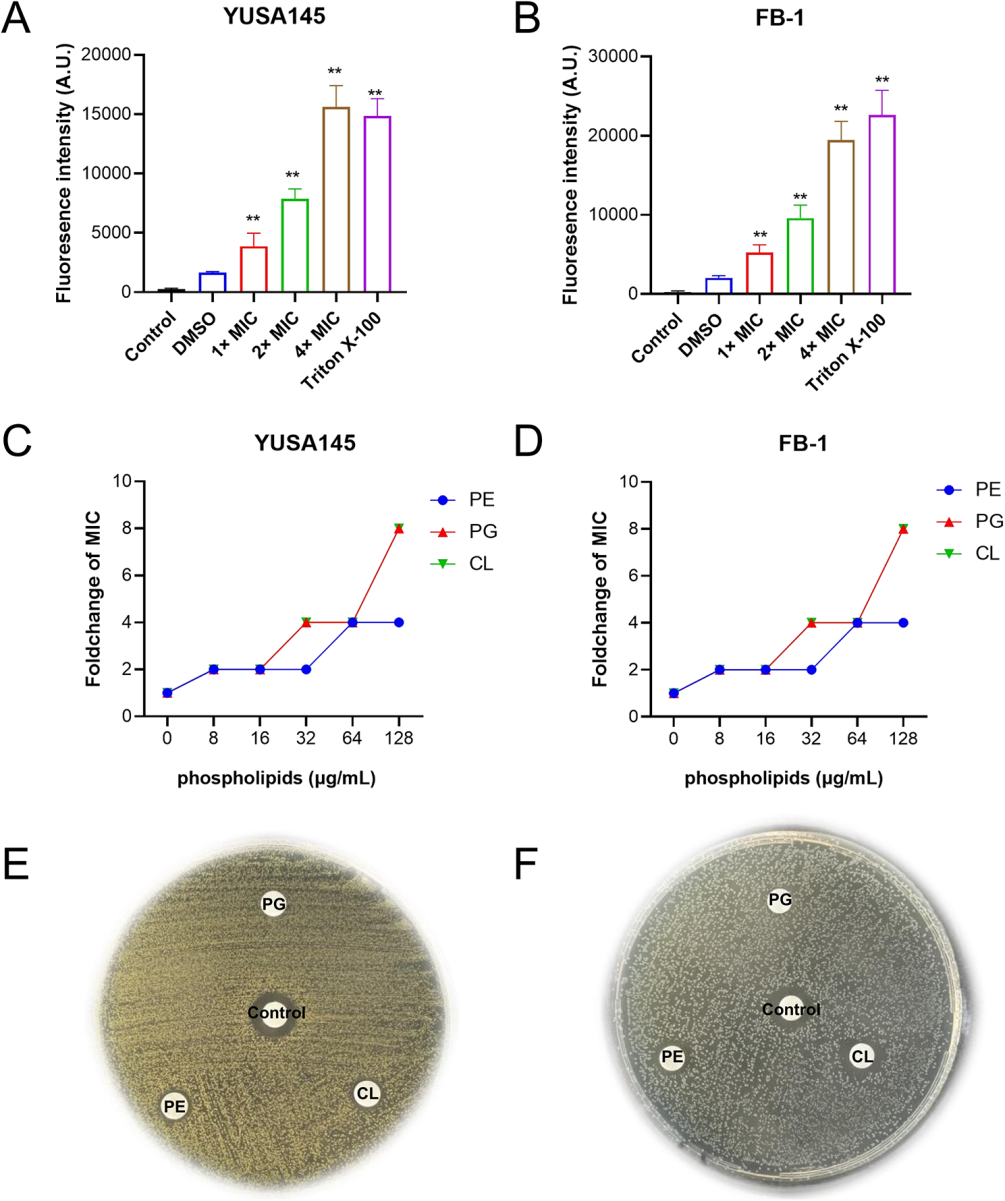
D-3263 targets bacterial membrane phospholipids. The effect of D-3263 on the membrane permeability of *S. aureus* (**A**) and *E. faecalis* (**B**). The DMSO group represents the solvent-treated group, while the control group denotes the negative control consisting of PI and D-3263 without bacterial cells. The data were presented as the average of three independent experiments (mean ± SEM). Statistical significance was determined using Student’s *t*-test, where ** indicates *P* < 0.01 compared to the DMSO group. Joint effect of D-3263 and different membrane phospholipid components on clinical isolated MRSA YUSA145 (**C**) and *E. faecalis* FB-1 (**D**). Three types of phospholipids including phosphatidylethanolamine (PE), phosphatidylglycerol (PG), and cardiolipin (CL) were used, with concentrations ranging from 0 to 128 µg/mL. Exogenous addition of phospholipids abolishes the antibacterial activity of D-3263 against *S. aureus* (**E**) and *E. faecalis* (**F**) evaluated by Kirby-Bauer (K-B) disk method. Inhibition zones of mixtures of 5 mM D-3263 and 5 mg/mL PE, PG and CL were recorded after incubated at 37 °C overnight

Due to the presence of multiple differentially expressed proteins in the cell membrane following D-3263 treatment as indicated by the proteomic results, as well as significant KEGG pathways enriched in *S. aureus* infection by differentially expressed proteins were all secreted proteins. Specifically, the presence of proteins involved in secretion pathways may indicate alterations in membrane integrity. Additionally, considering that structurally similar compounds have been shown to disrupt bacterial cytoplasmic membranes [21], we hypothesized that the antimicrobial mechanism of D-3263 involved in targeting the bacterial cell membrane to disrupt bacterial integrity. Firstly, the fluorescence dye propidium iodide (PI) was used to detect changes in membrane permeability of *S. aureus* and *E. faecalis* after D3263 treatment (Fig. 5A-B). These results suggested that treatment with D3263 significantly increased bacterial membrane permeability. Checkerboard assays were conducted to evaluate the impact of phospholipids on the antibacterial activity of D-3263. Three common bacterial membrane phospholipids, namely phosphatidylethanolamine (PE), phosphatidylglycerol (PG) and cardiolipin (CL) [22], were selected for this assay. They were combined with D-3263 and tested against clinically isolated strains of MRSA YUSA145 and *E. faecalis* FB-1. The resulting MICs of D-3263 were measured after 20 h and recorded (Fig. 5C-D). As the concentration of phospholipids increased from 0 to 128 µg/mL, the fold change in MIC of D-3263 also increased. Specifically, it increased to 8-fold with PG and CL and to 4-fold with PE, for both isolates. This upward trend in D-3263 MIC with higher phospholipid concentration indicated that phospholipids had an inhibitory effect on the antibacterial activities of D-3263. Consistent with that, exogenous addition of PG, CL, and PE dramatically decreased the inhibitory zone of D-3263 in the Kirby-Bauer (K-B) disk, as shown in Fig. 5E and F. These suggested that the bacterial cell membrane might be a potential target of D-3263, contributing to its antibiotic effects on Gram-positive bacteria.

## Discussion

Recently, the emergence of multidrug-resistant strains of *S. aureus* has posed a significant challenge in healthcare settings. These strains not only exhibit resistance to methicillin but also to last-resort antibiotics such as vancomycin, linezolid, and daptomycin, highlighting the critical need for novel antibiotics with unique mechanisms of action [23]. In this study, we revealed the antibacterial properties of the TRMP8 agonist D-3263 [19]. While previous research primarily focused on its role in inducing apoptosis in cancer cells, our findings demonstrated its potential as an antibiotic. D-3263 exhibited robust antibacterial activity against Gram-positive bacteria, including *S. aureus*, *E. faecalis*, and *E. faecium*, with MICs of ≤ 25 µM. Moreover, D-3263 not only inhibited bacterial growth but also displayed significant bactericidal effects, particularly at concentrations 4× and 8× MIC. Biofilm formation is a critical virulence factor that complicates treatment by promoting chronic infections. D-3263 demonstrated its efficacy in both preventing biofilm formation and eradicating mature biofilms in *S. aureus* and *E. faecalis*. These comprehensive antibacterial and antibiofilm activities positioned D-3263 as a promising candidate for combating chronic, multidrug-resistant *S. aureus* infections. However, it’s essential to note that the research on D-3263 is still in the early stages, and its safety profile, toxicity, and optimal dosage for human use have not been fully explored as it awaits FDA approval [24]. Further studies and clinical trials are warranted to assess its suitability for clinical applications.

We proceeded to employ quantitative proteomics to delve deeper into the mechanisms underpinning D-3263’s antibacterial prowess. Utilizing advanced mass spectrometry technology, we scrutinized the global proteomic response of MRSA YUSA145 when subjected to D-3263 stress. This comprehensive dataset offered valuable insights into the potential targets and pathways through which D-3263 exerted antibacterial effects. Our findings revealed that D-3263 exerted a multifaceted impact on the metabolic pathways of *S. aureus*. For instance, choline dehydrogenase betA, responsible for converting choline into the osmoprotectant glycine betaine, was down-regulated in the D-3263 treated group. Glycine betaine plays a crucial role in various metabolic processes, particularly in methylation and amino acid metabolism. The down-regulation of betA suggested that D-3263 disrupted choline and glycine metabolism, potentially affecting vital cellular processes [25–27]. We also observed an up-regulation of groES, a chaperonin protein that collaborates with groEL to facilitate protein folding. The groES/groEL chaperonin system is known to interact with a multitude of proteins in *Escherichia coli*, with more than 50 proteins relying on this system for proper folding [28]. Although most research on groES/groEL is based on *E. coli*, the conservation of sequences and structures implies that this system plays a pivotal role in all organisms, including *S. aureus* [29, 30]. The increase in groES expression suggested that D-3263 disrupted amino acid and protein synthesis in *S. aureus*, as supported by the up-regulation of ribosomal proteins rpsF and rpmE2. It was plausible that this disruption affected certain protein products vital for bacterial survival, contributing to the antibacterial effects of D-3263. Furthermore, our KEGG pathway analysis highlighted the significant enrichment of *S. aureus* infection-related pathways. Virulence factors such as γ-hemolysin hlgB and immunoglobulin (IgG) binding protein SBI exhibited differential expression, potentially linked to D-3263’s impact on *S. aureus* virulence. The signal transduction protein traP, a major regulator of staphylococcal pathogenesis, was also differentially expressed under D-3263 stress [31]. Phosphorylation of traP leads to the activation of the *accessory gene regulator* (*agr*) system and subsequent synthesis of *RNAIII*, pivotal in controlling quorum sensing, secretion, surface attachment, and biofilm formation in *S. aureus* [32, 33]. The up-regulation of traP suggested that *S. aureus* may employed a protective mechanism in response to environmental stress induced by D-3263. The intricate interplay between D-3263, this signal transduction protein, and the downstream proteins it regulated may contribute to D-3263’s anti-biofilm properties. In summary, our proteomic analysis provided valuable insights into the multifaceted mechanisms through which D-3263 exerted antibacterial effects.

Unraveling the direct target and mechanisms underlying the action of antibacterial compounds serve two crucial purposes. Firstly, it aids in the development of improved derivatives with enhanced therapeutic efficacy. Secondly, it opens up new target for the development of novel antibacterial agents. Gram-positive bacterial cell membranes predominantly consist of key lipid components, including phosphatidylethanolamine (PE), phosphatidylglycerol (PG), and cardiolipin (CL) [22, 34]. Among these, PG and CL are the major phospholipids in *S. aureus* cells. Antibacterial molecules targeting bacterial phospholipids represent a promising avenue in drug discovery. For instance, daptomycin is an important antibiotic that interacts with the bacterial membrane lipid PG in a calcium-dependent manner, leading to membrane destabilization and cell death [35]. The newly discovered variety of lipid-targeting antimicrobial agents such as AMXT-1501 and natural flavonoids α-mangostin and isobavachalcone, have the capability to overcome existing resistance barriers posed by resistant genes [36, 37]. In our study, we made an intriguing observation of a dose-dependent reduction in the antibacterial activity of D-3263 when exposed to the phospholipid components of bacterial cell membranes. The addition of PE, PG, and CL resulted in a substantial increase in the MIC of D-3263 for both *S. aureus* and *E. faecalis*, elevating it up to eight times the original MIC observed without the additional phospholipids. Furthermore, D-3263 increased bacterial membrane permeability. These findings strongly suggested that D-3263 might exert its action on the bacterial cell membrane. Further investigations in the future may delve deeper into elucidating the specific mechanisms and specific target sites within the bacterial cell membrane affected by D-3263 [38].

## Conclusion

In summary, this study represented the first comprehensive exploration of D-3263’s remarkable antibacterial properties against Gram-positive bacteria, including *S. aureus* (both MSSA and MRSA), *E. faecalis* and *E. faecium*. D-3263 exhibited exceptional antibiofilm capabilities, encompassing the inhibition of biofilm formation, eradication of mature biofilms. Our investigation into the action mechanism of D-3263, utilizing MS-based proteomics, suggested that its mode of action likely involved targeting the bacterial cell membrane and disrupting protein synthesis. As we move forward, future research endeavors should be directed towards identifying the specific antibacterial targets of D-3263. Additionally, gaining a deeper understanding of D-3263’s pharmacological properties and its potential toxicity in the human body would be crucial steps in assessing its suitability for clinical use. These insights had the potential to pave the way for the development of innovative antibacterial therapies and contribute to our ongoing battle against antibiotic-resistant pathogens.

## Methods

### Bacterial strains and growth conditions

The study involved a total of 30 clinical isolates of *S. aureus*, comprising 15 MSSA and 15 MRSA isolates. Additionally, 10 clinically isolated strains each of *E. faecalis* and *E. faecium* were included. These isolates were collected from the Huazhong University of Science and Technology Union Shenzhen Hospital over a period spanning from January 1, 2015, to December 31, 2018. Initial genus and species identification of the clinical isolates was performed using the Phoenix 100 automated microbiology system (BD, Franklin Lakes, NJ, USA), with subsequent confirmation through matrix-assisted laser desorption ionization time-of-flight mass spectrometry (IVD MALDI Biotyper, Germany) upon subculture. All procedures involving human subjects were approved by the institutional ethical committee of Huazhong University of Science and Technology Union Shenzhen Hospital and were conducted in accordance with the principles outlined in the 1964 Helsinki Declaration and its subsequent amendments. Isolates were collected as part of the routine clinical management of patients, according to the national guidelines in China. Therefore, informed consent was not sought. For control purposes, standard strains of *S. aureus*, including ATCC 29,213 and USA300, as well as standard strains of *E. faecalis*, including ATCC 29,212 and OG1RF, were procured from the American Type Culture Collection (ATCC). D-3263 hydrochloride, the compound of interest, was sourced from MedChemExpress (MCE, Shanghai, China).

### Minimal inhibitory concentration (MIC) test

The MICs of D-3263 and linezolid against *S. aureus, E. faecalis* and *E. faecium* were measured by broth dilution method, similar to our previous studies, and repeated in triplets [39]. Overnight cultures were adjusted to 0.5 McFarland turbidity, then diluted at 1:200 proportion with cation-adjusted Mueller Hinton broth (CAMHB, Huankai, Guangdong, China). Cultures were added to 96-well plates with descending concentrations of the drug. The MIC was defined as the lowest concentration of drug that had bacteria growth with optical density at 600 nm (OD_600_) ≤ 0.1 after 18 h of incubation, determined by the VITEK 2 system (bioMérieux, Marcyl’Etoile, France). The MIC results for antibiotics were interpreted according to the Clinical and Laboratory Standards Institute (CLSI) breakpoints, which served as a reference standard for evaluating antibiotic susceptibility.

### Growth curve analyses

For both *S. aureus* and *E. faecalis*, overnight cultures were initially diluted at a 1:1000 ratio using TSB (Tryptone Soy Broth, Huankai, Guangdong, China). These diluted cultures were then exposed to different concentrations of D-3263, specifically at 1×, 1/2×, 1/4×, and 1/8×MIC. To establish a control group for comparison, a set of cultures without the addition of any drug was prepared as well. All of these prepared cultures were placed in an automatic growth curve analyzer, consistent with previous reports [40]. These cultures were maintained at a temperature of 37 °C and subjected to shaking at a rate of 200 rpm. To determine planktonic cell growth, the optical density at 600 nm (OD_600_) was measured for each well at 60-minute intervals over a period of 24 h. This data collection allowed for the assessment of the effects of D-3263 on planktonic cell growth.

### Time killing assay

To assess the bactericidal effect of D-3263 on planktonic cells during their logarithmic growth phase, a time-kill assay was conducted.=The planktonic cells were treated with either D-3263 or linezolid, resulting in final concentrations of 2× MIC, 4× MIC, or 8× MIC. Samples were collected and analyzed at different time points: 0, 3, 6, 9, and 24 h. For each sampling, 1 mL of the bacterial culture was collected and then centrifuged at 8000 rpm for 3 min to separate the bacterial cells from the TSB. Following centrifugation, the bacterial cells were serially diluted with Mueller-Hinton broth, and 5 µL aliquots were plated onto TSB agar plates. These plates were then incubated at 37 °C for 24 h. The viable cells were subsequently quantified by counting colony-forming units (CFUs). This assay allowed for the assessment of the bactericidal activity of D-3263 against the tested strains over time.

### Biofilm analysis by crystal violet straining

This assay was conducted by adding 100 µL of overnight cultures of *S. aureus* or *E. faecalis* 1:500 diluted with TSBG (Tryptone soy broth with 2% glucose, in accordance with our previous studies [41]) into the 96-well plates, with 100 µL of descending concentration of D-3263. Final concentrations of D-3263 at 1/2×, 1/4×, and 1/8×MIC were used, as well as a negative control with TSBG only and a positive control with DMSO only. The wells were gently washed three times with 200 µL of Phosphate Buffer Saline (PBS), followed by drying in an inverted position. Subsequently, the wells were stained with 1% crystal violet (Thermo Fisher Scientific, Ohio, USA) for 15 min. The crystal violet was solubilized in ethanol-acetone (80:20, V/V). After staining, the wells were rinsed three times with PBS again. The amount of biofilm was quantified by measuring the optical density at 570 nm (OD_570_). This experiment was performed in triplicate at least three independent times.

### Mature biofilm eradication

Overnight cultivated strains of *S. aureus* and *E. faecalis* were 1:500 diluted with TSBG and added to 96-well plates. These cultures were then incubated at 37 °C for 24 h without the addition of any drugs. This allowed for the maturation of biofilms.After the initial 24-hour incubation period, the upper supernatant was replaced with fresh TSBG containing varying concentrations of D-3263 (1×, 2×, 4×, and 8× MIC). The cultures were then incubated again at 37 °C for an additional 24 h. Following this second incubation period, the remaining biomass of the biofilm was quantified using the crystal violet staining method, and the OD_570_ was measured.

### Sample preparation for quantitative proteomics

The clinical isolated MRSA strain YUSA145 was cultured to the exponential growth phase with an OD_600_ of 0.5 in TSB. The culture was then divided into two groups: the control group and the group treated with 1/2× MIC D-3263. Each group had four replicates. Both groups were further cultured for an additional 2 h at 200 rpm. After this incubation, the microbial cells were centrifuged at 12,000 rpm for 10 min. They were then washed twice with PBS buffer and stored at -80 °C until further protein extraction.To extract proteins, the cell pellets were suspended in RIPA lysis buffer, which contained a complete protease inhibitor cocktail. The cells underwent three rounds of homogenization. After homogenization, the samples were centrifuged at 12,000 rpm for 30 min at 4 °C, and the supernatants were collected for protein concentration determination using a BCA protein assay kit. Next, 100 µg of protein was prepared, reduced with 10 mM DTT (Dithiothreitol), and then alkylated using 50 mM iodoacetamide. The proteins were then desalted and digested with trypsin overnight for further analysis.

### Nano LC–MS/MS analysis

The prepared proteins were resuspended in 30 µL of 0.1% formic acid. Then, 4 µg of protein was injected into an LC (Liquid Chromatography) system. This LC system consisted of an UltiMate 3000 RSLC nano system and utilized a C18 precolumn. Separation was achieved using a C18 tip column with dimensions of 75 μm × 250 mm and a particle size of 2 μm. The LC system was coupled to a Q Exactive Plus mass spectrometer, which was equipped with a Nano spray ionization (NSI) interface. For the mass spectrometry analysis, MS1 scans were performed over a mass range of 300–1500 m / z (mass-to-charge ratio) with a high resolution of 70,000. MS2 spectra were acquired at a resolution of 17 500 and were collected for a maximum duration of 50 ms. All multiply charged ions were used to trigger MS-MS scans, followed by a dynamic exclusion for 30 s. Singly charged precursor ions and ions of undefinable charged states were excluded from fragmentation.

### Bioinformatics analysis for quantitative proteomics

Proteome Discoverer 2.4 based with Sequest HT was used for protein identification and quantification, conducted against the Uniprot proteome of *S. aureus* (strain NCTC 8325 / PS 47). Up-regulated proteins and down-regulated proteins were decided by calculating the *p*-value < 0.05 in at least two replicates and a 2-fold cut-off value. Gene ontology (GO) annotation applied for differentially expressed proteins was done with the omicsbean online database.

### Membrane permeability assay

The membrane disruption was identified by propidium iodide (PI) staining. MRSA isolate YUSA145 and *E. faecalis* FB-1 in exponential growth phase were gathered by centrifugation and adapted to OD_600_ of 0.05 and incubated with 2 µM PI and HEPES buffer (5 mM, pH 7.2) at dark. After incubation, the suspension cells were then treated with 1 × MIC, 2× MIC and 4× MIC D-3263. Different concentrations of D-3263 were dissolved in 1% DMSO, and solvent 1% DMSO and 0.1% Triton in PBS were added as control. A group containing only PI and treated with 4× MIC of D-3263 but without bacteria serves as the negative control. The fluorescence intensities were monitored in black polystyrene microtiter plates after 30 min by Cytation 5 cell imaging multi-mode reader (BioTek, Winooski, VT, USA) at an excitation wavelength of 535 nm and an emission wavelength of 615 nm.

### Checkerboard

MRSA YUSA145 and *E. faecalis* FB-1 were first cultivated to the logarithmic growth phase. Then, they were diluted 1:1000 with TSB and added to 96-well plates. D-3263 was added at a concentration of 400 µM in the first row of the plate, and the concentration was decreased in descending order down the rows. Various phospholipids, such as phosphatidylglycerol (PG, Aladdin, China), Phosphatidylethanolamine (Y0001953, Sigma–Aldrich, USA), or cardiolipin (CL, Sigma–Aldrich, C0563, USA), were dissolved in methanol. Each type of phospholipid (PE, PG, and CL) was added at a concentration of 128 µg/mL in the first column of the plate, and the concentration was decreased in descending order across the columns. The isolates in the plate were then incubated at 37 °C for 18 h. After incubation, the MIC results were determined as mentioned earlier.

### K-B disk diffusion method

After overnight, the culture was diluted 1000-fold with PBS and then spread onto CAMHB plates using sterile cotton swabs. Next, sterile filter disks were placed on the plates. 10 µL of 5 mM D3263 was dropped onto filter disks as a control. For the phospholipid treatment group, 10 µL of 5 mM D3263 were mixed with 10 µL of 5 mg/mL PG, PE, or CL, respectively, and then dropped onto filter disks. After 24 h of incubation at 37 °C, the results were photographed and recorded.

### Statistical analysis

Graphpad prism 8.0 software was used to process data and draw images. Comparisons of differences in biofilm formation, the membrane permeability and CFU between the control group and the D-3263-treated group were analyzed using Student’s t-test. *P* < 0.05 was considered as statistically significant.

### Electronic supplementary material

Below is the link to the electronic supplementary material.

Supplementary Material 1

**Fig. S1** PCA (A) and heatmap analysis (B) of global differences in the protein profiles between the D-3263 treatment group and the control group.

## Data Availability

The mass spectrometry proteomics data have been deposited to the ProteomeXchange Consortium via the iProX partner repository with the dataset identifier PXD046347.

## Abbreviations

TRPM8: Transient receptor potential melastatin member 8
MIC: Minimum inhibitory concentration
MRSA: Methicillin-resistant S. aureus
PE: Phospholipids phosphatidylethanolamine
PG: Phosphatidylglycerol
CL: Cardiolipin
PBP 2a: Penicillin-binding protein 2a
MSSA: Methicillin-sensitive S. aureus
Ca^2+^: Calcium ion
GO: Gene Ontology
KEGG: Kyoto Encyclopedia of Genes and Genomes
PI: Propidium iodide
TSB: Tryptone soy broth
OD: Optical density
CFU: Colony-forming units
TSBG: Tryptone soy broth with 2% glucose
PBS: Phosphate Bufer Saline
DMSO: Dimethyl sulfoxide
MS: Mass spectrometry
