# Supplementary material for: Antibacterial activity and mechanisms of D-3263 against Staphylococcus aureus
Source: BMC Microbiol. 2024 Jun 26;24:224. doi: 10.1186/s12866-024-03377-3 (PMC11201875; doi:10.1186/s12866-024-03377-3)
Supplement: Supplementary file 1 — Supplementary Material 1 [file 12866_2024_3377_MOESM1_ESM.docx]

Table S1. MIC values of D-3263 toward Gram-negative bacteria

| **Species** | **isolate** | **MIC (μM)** |
| --- | --- | --- |
| *Acinetobacter baumannii* | *AB1* | ＞200 |
| *Klebsiella pneumoniae* | *K2044* | ＞200 |
| *Escherichia coli* | *ATCC25922* | ＞200 |
| *Pseudomonas aeruginosa* | *ATCC27853* | ＞200 |
