# Supplementary figures and images for: Antibacterial activity and mechanisms of D-3263 against Staphylococcus aureus
Source: BMC Microbiol. 2024 Jun 26;24:224. doi: 10.1186/s12866-024-03377-3 (PMC11201875; doi:10.1186/s12866-024-03377-3)

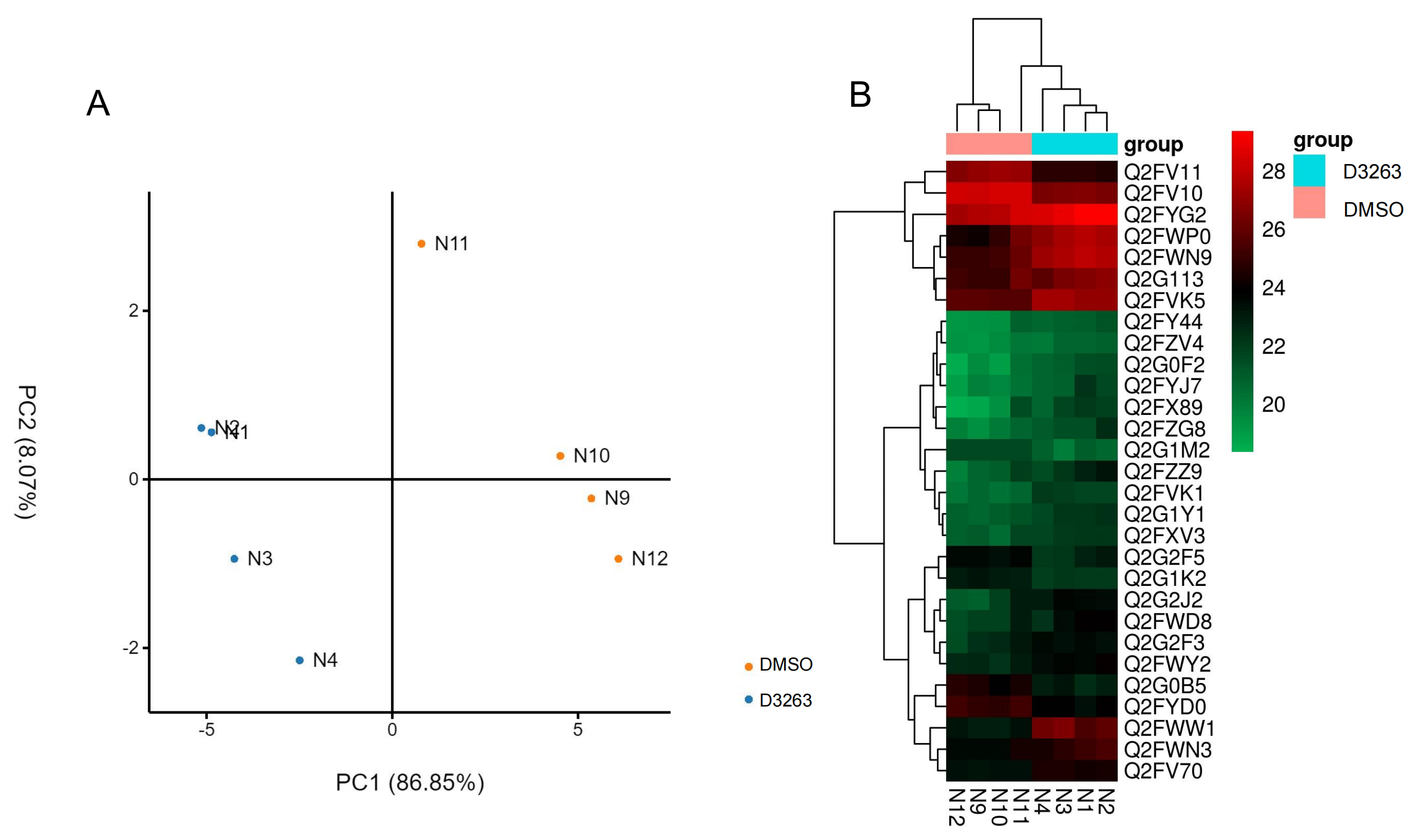

Supplement: Supplementary file 2 — Fig. S1 PCA (A) and heatmap analysis (B) of global differences in the protein profiles between the D-3263 treatment group and the control group. [file 12866_2024_3377_MOESM2_ESM.tif]
